# Primary pulmonary amyloidosis misdiagnosed as malignancy on dual-time-point fluoro-deoxyglucose positron emission tomography/computed tomography: A case report and review of the literature

**DOI:** 10.3892/ol.2014.2778

**Published:** 2014-12-08

**Authors:** MENG-JIE DONG, KUI ZHAO, ZHEN-FENG LIU, GUO-LIN WANG, JUN YANG

**Affiliations:** 1 Positron Emission Tomography Center, First Affiliated Hospital, College of Medicine, Zhejiang University, Hangzhou, Zhejiang 310003, P.R. China; 2 Department of Nuclear Medicine, First Affiliated Hospital, College of Medicine, Zhejiang University, Hangzhou, Zhejiang 310003, P.R. China

**Keywords:** 18F-fluoro-deoxyglucose positron emission tomography/computed tomography, pulmonary amyloidosis, lung malignancies

## Abstract

Primary pulmonary amyloidosis is an uncommon manifestation, characterized by amyloid deposition in the lungs and other associated tissue. The clinical presentation of amyloidosis is variable, with non-specific symptoms. The current study reports the case of a 59-year-old female presenting with primary pulmonary amyloidosis, indistinguishable from lung malignancy based on 18F-fluoro-deoxyglucose (18F-FDG) accumulation on dual-time-point (DTP) FDG-positron emission tomography/computed tomography (PET/CT) imaging and the similarities in morphological changes. A percutaneous CT-guided thoracoscopic biopsy was subsequently performed. Histological examination revealed that the specimens contained amorphous, homogeneous material with a number of polyclonal plasma cells, lymphocytes and giant cells. A diagnosis of primary nodular parenchymal pulmonary amyloidosis was determined, and the patient was discharged without chemotherapy. The patient remained in good clinical condition during follow-up. The present case indicated that localized nodular amyloidosis with increased FDG uptake on DTP FDG PET must be considered in the differential diagnosis of growing lung nodules, and that a histological examination must be conducted to distinguish this condition from malignancies of the lung.

## Introduction

Primary pulmonary amyloidosis is an uncommon disease, characterized by amyloid deposits localized to the respiratory system (1,2). Respiratory amyloidosis was first described in 1877 by Lesser (3). Although the number of reported cases has accumulated over past years, the exact pathogenesis of the disease remains unclear. It is believed that the misfolding of extracellular protein plays a prominent role in the molecular mechanism of amyloidosis. Respiratory impairment is uncommon and can be classified as three forms: Diffuse interstitial deposits, single or multiple pulmonary nodules and submucosal tracheobronchial deposits (4). Amyloid deposition in the tracheobronchial airways is rare and constitutes ~1% of benign tumors (5,6), with only 12 cases of solitary thoracic amyloidomas reported in the literature, as reviewed by Cresner *et al* (7). The natural history of this disorder and the efficacy of potential therapies have not been clearly defined. Thorough evaluation of respiratory tract amyloidosis is required to determine the type and need for treatment. Broadly, systemic chemotherapy is indicated for systemic amyloidosis and local intervention for its localised forms. The management of tracheobronchial amyloidosis is also largely dependent upon symptoms and may involve intermittent bronchoscopic resection, surgical resection, carbon dioxide laser ablation and neodymium-doped yttrium, aluminum and garnet laser therapy (7). The manifestations, clinical significance and prognosis of respiratory tract amyloidosis vary considerably depending on its etiology and anatomical distribution. Therefore, each patient requires thorough evaluation to determine their optimal management (8). The clinical presentation of pulmonary amyloidosis varies between cases and the symptoms are non-specific, causing diagnosis to be problematic. The predominant differential diagnoses based on imaging are lung cancer, lung metastasis, tuberculoma and cryptococcosis. The ability to differentiate pulmonary amyloidosis from other disorders by diagnostic imaging as an alternative to invasive tests, such as bronchoscopy and biopsy under CT guidance, would be beneficial (4,9). Therefore, in the present study, biopsy under CT guidance was chosen, and shown to be effective.

Positron emission tomography (PET) with 18F-fluoro-deoxyglucose (18F-FDG) is widely used in the diagnosis of indeterminate solitary pulmonary nodules (SPNs) on computed tomography (CT) imaging (10). In particular, the standardized uptake values (SUVs) measured by dual-time-point (DTP) or delayed PET/CT imaging have been proposed to be helpful indicators in distinguishing malignant from benign SPN (11). To the best of our knowledge, only one reported case of pulmonary amyloid lesions has evaluated the disorder by DTP PET/CT imaging. Tan *et al* (12) presented a case of amyloidosis exhibiting significantly increased 18F-FDG accumulation in the right lung and the left lung lesions on the 1-hour early phase (maximum SUV was not shown). However, on the 2-h delayed images, compared with the levels of the 1-h images (maximum SUV was not shown), the metabolic activity of these lesions was markedly reduced. Therefore, it has been hypothesized that pulmonary amyloid lesions may potentially be distinguished from malignancy when utilizing dual phase FDG PET/CT imaging (12). Therefore, the dual phase FDG PET/CT imaging was performed in the present study.

The present study reports one case of primary pulmonary amyloidosis in a 59-year-old Chinese female, initially misdiagnosed as malignancy on DTP PET/CT imaging. The patient provided written informed consent for the study.

## Case report

A 59-year-old Chinese female was referred to the chest clinic of the First Affiliated Hospital, College of Medicine, Zhejiang University (Hangzhou, China) in April 2011 due to a two-month history of cough, hemoptysis and general fatigue. A CT scan of the chest revealed a nodule in the left lower lung with mild enhancement in the lesion. The nodule progressed during the two-month follow-up period under treatment with anti-infection drugs. No other significant history was noted. The patient’s physical examination was unremarkable, and hematological and biochemical parameters were within normal limits.

**Figure 1:**
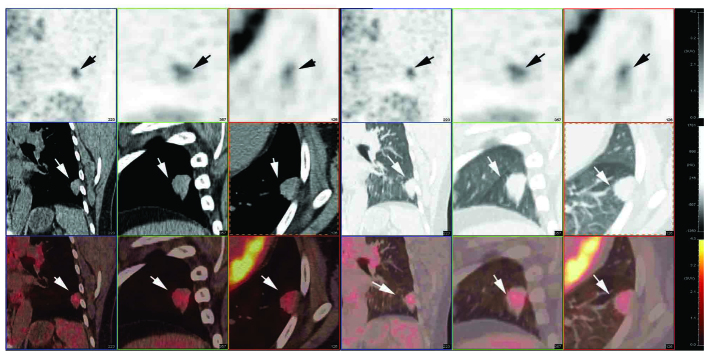
Primary pulmonary amyloidosis on 18F-FDG PET/CT. The PET/CT images revealed a 1.83×1.40 cm lobulated nodule with burr-like margins in the left lower lung exhibiting increased FDG uptake. Continuous slice images are shown from left to right (CT and PET/CT fusion images of the mediastinal window and lung window are shown, respectively). Upper row, PET images (arrows indicate lesions with increased FDG uptake); middle row, CT images (arrows indicate lobulated nodules with burr-like margins); lower row, PET/CT fusion images (arrows indicate lesions with increased FDG uptake in the lobulated nodule). FDG, fluoro-deoxyglucose; PET/CT, positron emission tomography/computed tomography.

**Figure 2:**
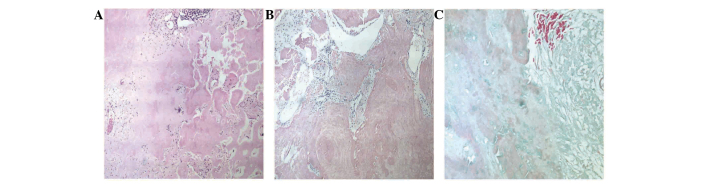
Histopathology of nodular amyloidosis in the primary lesions. (A) Polyclonal plasma cells, lymphocytes and giant cells surround the amorphous eosinophilic deposits (hematoxylin and eosin staining; magnification, ×100). Immunohistochemical studies showed (B) immunopositivity towards Congo red staining (magnification, ×400) and (C) immunonegativity towards trichrome staining (magnification, ×400).

A dual phase FDG PET/CT scan was performed following six hours of fasting. FDG (5.5 MBq/kg) was injected intravenously through an antecubital vein while the patient remained at rest. Image acquisition was subsequently conducted using a Siemens Biograph 16 PET-CT scanner (Siemens Medical Solutions USA, Inc., Malvern, PA, USA). The PET/CT images revealed a 1.83×1.40 cm slightly lobulated nodule, with burr-like margins in the left lower lung, exhibiting moderately increased F-18 FDG uptake (maximum SUV of 2.6) in the initial images (1 h following the FDG injection), and more intense FDG uptake with an SUV of 3.5 (26.9% increase) in the delayed images (2 h following the injection) (Fig. 1). Based on the dual phase FDG PET/CT imaging findings, morphological features, contrast-enhanced chest CT imaging and medical history (progression of the nodule during the two-month follow-up period), lung malignancy was highly suspected. A percutaneous CT-guided thoracoscopic biopsy was subsequently performed. Histological examination revealed that the specimens contained amorphous, homogeneous material with a number of polyclonal plasma cells, lymphocytes and giant cells. Eosinophilic material exhibited apple-green birefringence under polarizing microscopy. Immunohistochemically, congo red staining was positive, and trichrome staining was negative, confirming the deposition of amyloid within the specimen (Fig. 2). Therefore, a diagnosis of primary nodular parenchymal pulmonary amyloidosis was determined, and the patient was discharged without chemotherapy and other treatment. After May 2011, the patient was followed up every 3 months and was in good clinical condition at the time of writing.

## Discussion

Pulmonary localized nodular amyloidosis, characterized by the deposition of various proteins that form insoluble β-pleated sheets in the lung parenchyma, is a rare disorder and is not associated with primary systemic amyloidosis (1,2,13).

Integrated PET/CT provides both metabolic and morphological information for the characterization of nodules, and is rapidly becoming a front-line modality in the evaluation of SPNs (14). The diagnostic accuracy of FDG avidity has been demonstrated by numerous studies to be ~90% for the assessment of SPNs (15–17) and the application of DTP imaging in particular has been reported to potentially improve diagnostic accuracy. For differentiation between benign and malignant lesions in the thorax, Demura *et al* (18) estimated the sensitivity and specificity to be 74% [37/50; 95% confidence interval (CI), 0.60–0.85] and 50% (15/30; CI, 0.31–0.69), respectively, for initial imaging, and 98% (49/50; CI, 0.89–1.00) and 67% (20/30; CI, 0.47–0.83), respectively, for DTP imaging in 80 patients with thoracic nodular lesions. The authors concluded that DTP imaging was more accurate compared with single-time-point scanning, with the exception of use in patients with active granulomatous diseases. Matthies *et al* (19) reported 36 patients with 38 known or suspected malignant pulmonary nodules who underwent PET of the thorax at two time points: Scan one at 70 min (range, 56–110 min) and scan two at 123 min (range, 100–163 min) after the intravenous injection of 2.5 MBq 18F-FDG per kilogram of body weight. The sensitivity and specificity were found to be 80% (16/20; CI, 0.56–0.94) and 94% (17/18; CI, 0.73–1.00), respectively, for initial imaging, and 100% (20/20; CI, 0.83–1.00) and 89% (16/18; CI, 0.65–0.99) for DTP imaging in the detection of malignant lung tumors. Though there are differences in the sensitivity and specificity data between these two studies, which may be due to the different sample sizes, it has been suggested that DTP FDG PET/CT imaging was high value in the diagnosis or differentiation between malignancy and benign for the patients with the SPNs.

To the best of our knowledge, few studies have reported the evaluation of pulmonary amyloid lesions by FDG PET, and each case has exhibited different FDG uptake activity (9–1§). Ishii *et al* (9) reported a nodular shadow in the right middle lobe with no FDG uptake observed on FDG PET imaging. The nodule was circular and smooth, with clearly demarcated borders, indicating benign nodules, which is significantly different from the present case. Benign nodules are generally well-defined and have smooth margins, whereas the majority of malignant SPNs have irregular and spiculated margins (20). Zhang *et al* (21) reported a 44-year-old male with different sized nodules in both lungs detected on a chest CT scan. As metastases were suspected in the multiple lung nodules, FDG PET/CT was conducted to characterize the nodules and to detect a possible primary malignancy. The PET/CT revealed that the nodules had a mild uptake of 18F-FDG suggestive of malignancy, with a maximum SUV of 1.19. Khan *et al* (22) and Soussan *et al* (23) revealed intense tracheobronchial FDG uptake (maximum SUV of 4.6) associated with an intense uptake in mediastinal fat, particularly in the surrounding aorta, which made it difficult to differentiate between malignanct and benign disease. DTP FDG PET/CT imaging may be an effective modality in the evaluation of these lesions and, thus, it was performed in the present study. However, only one case of a pulmonary amyloid lesion has been reported with identification via DTP FDG PET/CT imaging. Tan *et al* (12) presented a case of amyloidosis exhibiting increased 18F-FDG accumulation upon PET imaging. The 2-h delayed images revealed significantly reduced metabolic activity in these lesions, and the authors concluded that pulmonary amyloid lesions can potentially be distinguished from malignancies using a FDG PET/CT scan. DTP FDG PET/CT imaging was suggested as a discriminator between benign and malignant diseases, with images being obtained 1 and 2 h after the administration of 18F-FDG. Malignant lesions showed a significant increase in SUV over time, and those benign lesions showed a decrease over time (11,24).

The present case demonstrates that nodular amyloidosis may be indistinguishable from tumors due to similarities in DTP 18F-FDG PET images and in morphological changes. The DTP FDG PET scan revealed high FDG uptake in the initial images, and more intense FDG uptake (an increase of 26.9%) in the delayed images, which is consistent with the FDG uptake characteristics of malignancy (13). Morphological evaluations can aid in the differentiation between benign and malignant nodules only when they have typically benign or malignant features. Determining the growth rate of lung nodules by comparing current and prior CT images is an important and cost-effective step in the evaluation of SPNs (8). SPNs usually grow at constant rates, expressed as the doubling time. A nodule with a doubling time between 20 and 400 days is usually malignant, whereas benign nodules usually have a doubling time of >400 days (25). In the present case, a lobulated nodule with burr-like margins was discovered in the left lower lung, and exhibited progression during the two-month follow-up period. Based on the DTP FDG PET/CT imaging findings, morphological features, and medical history, lung malignancy was highly suspected, however, histological evaluation revealed that this diagnosis was incorrect and confirmed the lesions to be a result of pulmonary amyloidosis.

In conclusion, this case study indicates that localized nodular amyloidosis with increased FDG uptake on DTP FDG PET imaging must be considered during the differential diagnosis of growing lung nodules, and that a histological examination must be performed to distinguish this disorder from lung malignancies. Further prospective investigations on a larger sample of cases are required to better define the potential benefits of DTP 18F-FDG PET imaging in the diagnosis of primary pulmonary amyloidosis.
